# Analytical and Numerical Approaches for the Design of Concrete Structural Elements with Internal BFRP Reinforcement

**DOI:** 10.3390/ma15041497

**Published:** 2022-02-17

**Authors:** Todor Zhelyazov, Eythor Thorhallsson

**Affiliations:** Structural Engineering and Composites Laboratory (SEL), Reykjavik University, Menntavegur 1, IS-102 Reykjavik, Iceland; eythor@ru.is

**Keywords:** BFRP, BFRP-reinforced concrete beams, concrete, finite element modeling, pre-tensioning

## Abstract

Although basalt fiber-reinforced polymers (BFRPs) have been known for a few decades, new trends such as sustainability and environmental care have provoked intensified research on its structural applications. In construction, BFRPs, as internal reinforcement, have to compete with traditional steel reinforcement products. Because of their high resistance to aggressive environments, BFRPs have emerged as an attractive solution for the infrastructure in coastal zones. In this article, we discuss some aspects of BFRP applications such as flexural reinforcement of concrete beams. The mechanical performances of a BFRP-reinforced beam are illustrated by using a widely accepted model based on the classical beam theory. The elasticity modulus of the BFRP reinforcement is lower than that of structural steel. Therefore, to meet serviceability requirements (e.g., in terms of limitation on the mid-span deflection of a beam), BFRP could be pre-tensioned. The positive effect of pre-tensioning is outlined by finite element analysis. An original numerical procedure involves a constitutive relation for concrete based on damage mechanics. Experimental results previously reported in the literature provide the background for the numerical model procedures. The numerical procedure predicts the mechanical response of the concrete beam with BFRP reinforcement subjected to four-point bending in terms of load-deflection relationship and dominant failure mode.

## 1. Introduction

As compared with steel, fiber-reinforced polymers (FRPs) are approximately three times lighter and have five times higher tensile strength [1,2,3,4]. Among the other currently used FRP such as carbon FRP (CFRP), glass FRP (GFRP), and aramid FRP (AFRP), basalt FRP (BFRP) is a relatively new option for reinforcement or strengthening of structural elements. BFRP materials have excellent fatigue resistance [5], they are eco-friendly, noncorrosive, nontoxic, and have good magnetic insulation properties [6]. Moreover, they are resistant to high temperature and high moisture conditions [6,7], and they are chemically stable [8,9]. Experimental investigations have shown that BFRPs have good performance when subjected to accelerated weathering tests. However, exposure to an alkali environment results in insufficient strength [10]. Research works have reported that BFRPs have excellent freezing-and-thawing resistance [11] and good resistance to hostile acidic environments [12].

BFRP reinforcement has emerged as a potential replacement for traditional internal steel reinforcement, specifically for concrete structures in coastal zones. The replacement of steel with BFRP will possibly reduce structural deterioration due to corrosion [13,14]. Pilot projects (see, for example [15]) have revealed successful implementation of FRP (BFRP and GFRP) reinforcement for the rehabilitation of reinforced concrete structures in extremely aggressive environments. 

Despite all the above advantages, when applying BFRPs in their current form, designers have to circumvent some issues inherent in this type of reinforcement: (i) The elasticity modulus of BFRP is considerably lower than that of steel. Therefore, BFRP-reinforced beams provide lower flexural rigidity as compared with reinforced concrete beams. To satisfy serviceability requirements (e.g., in terms of mid-span deflection), the load-bearing capacity of a BFRP-reinforced beam should be limited as compared with that of a steel-reinforced concrete beam (of the same geometry and the same amount of flexural reinforcement). The pre-tensioning of the tensile reinforcement is a viable option to address this issue, even though it implies mounting dedicated anchorage devices in BFRP-reinforced beams, thus, complicating their fabrication. (ii) Generally, the shear strength of beams with flexural FRP reinforcement is lower than that of beams with flexural steel reinforcement in the absence of shear reinforcement. The lower shear resistance of FRP-reinforced beams, mainly attributed to the much lower dowel action, implies more rigorous requirements for transverse reinforcement. (iii) In their current form, BFRP bars require a sand coating to improve bond strength (of the BFRP-concrete interface). Overall, bond behavior for various loading conditions, including cycling and sustained load, requires additional research to mitigate computational uncertainty in design.

BFRP reinforcement is gaining positions in FRP structures. However, without being embraced by design guidelines in some countries (e.g., the United States), reportedly, the use of BFRPs still lags behind other FRP materials [16]. Since both the physical and mechanical characteristics of the reinforcing bars in BFRP differ from those of steel reinforcement, the safe and efficient application of BFRP in the construction industry needs additional research.

In this study, we investigate the mechanical response of a reinforced concrete beam strengthened to flexure with internal BFRP reinforcement. The scope of the study is limited to the analytical and numerical aspects of the structure response description. However, the performed simulations use an input (geometrical and material properties) and benchmark empirical data from sources in the literature. An investigation of beam behavior implies taking into consideration multiple resisting and failure mechanisms. For this reason, a numerical algorithm allows for the implementation of customized constitutive relations, and failure criteria is used for the prediction of the beam response. This study also includes an analytical approach based on the classical beam theory (i.e., by assumption, the concrete member loaded in flexure is composed of longitudinal fibers which do not interact). The beam behavior is assessed quantitatively by obtaining the load-deflection relationship.

Quantitative algorithms available in the design codes are based on a model initially developed to predict the moment-deflection relationship of a reinforced concrete beam loaded to flexure [17]. Based on extensive experimental research, this model has been subsequently modified to extend its validity by defining a set of coefficients to account for the particularities in the BFRP reinforcement behavior within structural elements. Currently, models based on the classical beam theory account for: (i) The properties of the constituent materials. (ii) The concrete failure behavior, specifically, the cracking in concrete replacing the gross moment of inertia by the cracked moment of inertia. (iii) The deformed shape of the member, loaded in flexure. Among the drawbacks of BFRPs, as compared with steel reinforcement, is the lower flexural rigidity the former provides. This issue can be addressed either by a larger cross-sectional area of the flexural reinforcement or by its pre-tensioning. To the authors’ best knowledge, the aforementioned model does not integrate the feature of pre-tensioning. To fill this gap, a model is proposed that is based on the finite element method (FEM), which takes into consideration the pre-tensioning. Moreover, the FEM-based model more accurately captures concrete failure behavior-from crack initiation into the initially undamaged material to crack propagation. The numerical procedure, using FEM, also predicts the potential failure mode of the structural element. 

A widely accepted analytical approach is used along with an original numerical procedure based on the FEM to assess the mechanical response of BFRP-reinforced beams. The numerical procedure takes into consideration the interaction of many interacting mechanisms (behavior of concrete loaded in shear and flexure, behavior of steel in the stirrups, and response of the pre-tensioned BFRP tendons). The numerical algorithm uses a constitutive relation for concrete defined in the framework of continuum damage mechanics. A damage variable is plugged into the stress-strain relationship. In this context, reaching the pre-established critical value of the damage variable at a specified location corresponds to macroscopic crack initiation. Thus, the employed algorithm can also predict the crack pattern at the structural element failure. In recent publications reporting advanced FEM simulations, damage mechanics has been applied as part of a complex numerical analysis [18,19]. At the same time, implementation of damage mechanics in research works focused on BFRP-reinforced concrete is not very frequent. The approach used herein has been applied in the analysis of the mechanical behavior of reinforced concrete beams strengthened to flexure with externally bonded CFRP [20]. 

According to recent trends, in addition to the need to satisfy purely structural provisions, structures and constituent materials are expected to meet a broader range of requirements. In this context, a discussion on the environmental impact of the BFRP material, as compared with that of traditional steel reinforcement, is proposed to readers.

## 2. Materials and Methods

**Figure 1:**
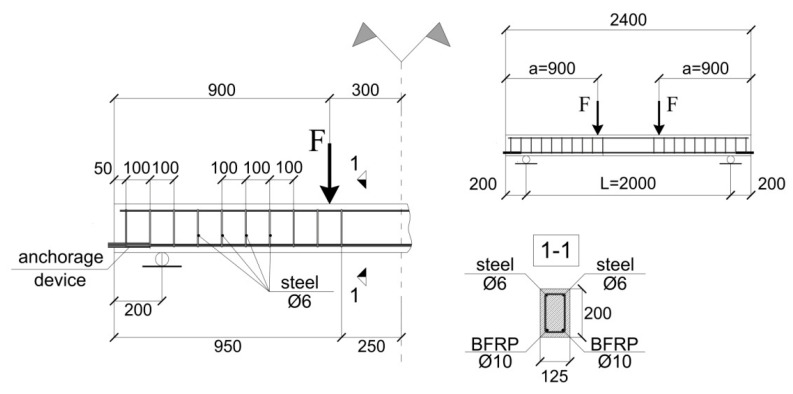
Geometry of the specimen and reinforcement arrangement [21].

**Figure 2:**
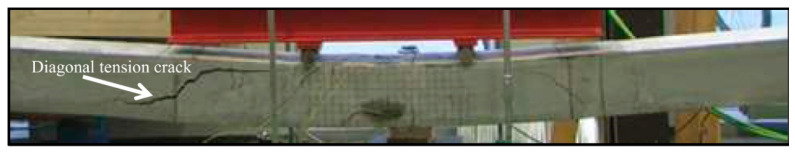
Concrete beam with tensile flexural BFRP reinforcement without shear reinforcement. Experimental setup for the four-point bending test [22].

This study reported herein is a continuation of previous experimental investigations of the mechanical response of concrete beams reinforced with tensile BFRP reinforcement and subjected to a four-point bending test, as shown in Figure 1 [21] and Figure 2 [22]. 

The failure of the beam shown in Figure 2, not equipped with stirrups, is identified with the unstable propagation of a diagonal tension crack resulting from insufficient shear resistance. As mentioned above, shear reinforcement in beams with flexural internal BFRP reinforcement is mandatory for practical applications. For this reason, the numerical study uses as input the geometry and the material properties of the beam shown in Figure 1. 

The modeled beam has a length of 2400 mm and a rectangular cross-section of 125 mm in width and 200 mm in height. The simply supported span and the constant moment region are 2000 mm and 600 mm, respectively. The internal tensile flexural reinforcement consists of two 10 mm diameter BFRP bars at the tension face. At the compression face, two 6 mm diameter mild steel bars are placed in order to support the steel stirrups. Between the beam end and the loading points, the stirrups are spaced at 100 mm, and between the loading points, no stirrups are placed. The concrete cover of 25 mm provides an effective depth of 170 mm. The pre-tensioned BFRP rods enhance the performances of the beam (flexural rigidity and load-carrying capacity).

A model based on the classical beam theory is employed to outline the effect of the type of internal flexural reinforcement (BFRP or mild steel) on beam response (in terms of the load–deflection relationship). This model, referred to further in the text as the “analytical model”, is currently available in some design code provisions. It has strong theoretical and experimental bases. Considerable research work has been carried out (see among others [23,24,25,26,27]) to identify the empirical coefficients introduced to account for the modalities of the fiber-reinforced polymers, regarded as constituencies of structural elements.

A detailed investigation of the BFRP-reinforced beam uses an original semi-analytical procedure based on the finite element method (referred to further as “finite element model”). The numerical algorithm takes into consideration the pre-tensioning of the BFRP reinforcement. It accounts for many interacting mechanisms (concrete shear and flexural resistance, contribution of stirrups, boundary conditions, and pre-tensioning) which depend on multiple factors. 

The following sections provide a discussion of the beam mechanical behavior considered in the context of each model. The discussion is supported by numerical results, some of which are compared with experimental data.

## 3. Analytical Model

Equation (1) provides an assessment of the deflection (Δ) of a concrete beam with longitudinal tensile BFRP reinforcement subjected to four-point bending [24] as follows:

(1) $$\Delta =\frac{{\alpha \mathrm{FL}}^{3}}{48E_{c}I_{e}^{\prime }}.$$

**Figure 3:**
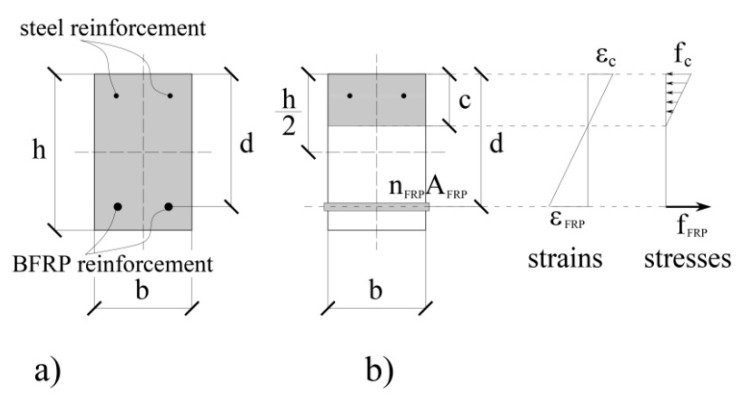
BFRP-reinforced beam: the active concrete cross-section (in grey) before (**a**) and after cracking (**b**); the strain and stress distributions (for the cracked cross-section) are schematized on the right.

In Equation (1), F stands for the applied load, L is the beam span, $I_{e}^{\prime }$ is the effective moment of inertia, Young’s modulus for concrete is taken as $E_{c}=4.73\sqrt{f_{c}^{\prime }}$ [28], where $f_{c}^{\prime }$ is the concrete compressive strength, and α is defined in Equation (2) as follows:

(2) $$\alpha =3\left(\frac{a}{L}\right)-4{\left(\frac{a}{L}\right)}^{3},$$

where a denotes the distance between the beam end and the load application point (Figure 1). The effective moment of inertia should be employed instead of the gross moment of inertia (or the moment of inertia of the uncracked) section when cracking in concrete occurs. The effective moment of inertia $\left(I_{e}\right)$ was developed to assure a transition between the upper and the lower bounds of the gross moment of inertia $\left(I_{g}\right)$, or the moment of inertia of the uncracked section (i.e., the whole concrete section still contributes to the flexural resistance of the beam (Figure 3a), ($I_{g}={\mathrm{bh}}^{2}/12$), and cracked moment of inertia ($I_{\mathrm{cr}}$) (defined according the scheme in Figure 3b). In actual guidelines [29], the effective moment of inertia can be estimated by using Expression (3):

(3) $$I_{e}={\left(\frac{M_{\mathrm{cr}}}{M_{a}}\right)}^{3}I_{g}+\left[1-{\left(\frac{M_{\mathrm{cr}}}{M_{a}}\right)}^{3}\right]I_{\mathrm{cr}}\leq I_{g}.$$

Presumably, cracking occurs when the bending moment resulting from the applied load ($M_{a}$) exceeds the cracking moment $\left(M_{\mathrm{cr}}\right)$, $M_{a}>M_{\mathrm{cr}}$. The cracking moment [28] is defined as:

(4) $$\;M_{\mathrm{cr}}=\frac{f_{r}I_{g}}{y_{t}}.$$

where $f_{r}$ is the modulus of rupture for concrete ($f_{r}=0.62\sqrt{f_{c}^{\prime }}\;$, $f_{c}^{\prime }$ being the concrete compressive strength obtained in characterization tests on standard cylindrical concrete specimens [28]), $y_{t}$ is the distance from the centroid of the cross section to the extreme tensile fiber. 

Recently, the accurate assessment of the effective moment of inertia of concrete beams with internal BFRP has drawn significant research interest [20,21,26]. Initially developed for beams with steel reinforcement, Equation (3) should be modified to accurately predict the load-deflection response of concrete beams with FRP reinforcement. Among the available empirical models, the one proposed by [24] is chosen because it integrates the effects due to the element’s curvature. In [24], the moment of inertia of the cracked section is approximated as follows:

(5) $$I_{e}^{\prime }=\frac{I_{\mathrm{cr}}}{1-\gamma \left(1-\frac{I_{\mathrm{cr}}}{I_{g}}\right){\left(\frac{M_{\mathrm{cr}}}{M_{a}}\right)}^{2}}\leq I_{g}.$$

In Equation (5), the coefficient γ accounts for the curvature. For a four-point bending, it is evaluated as follows:

(6) $$\gamma =\frac{3\left(a/L\right)-4\xi {\left(a/L\right)}^{3}}{3\left(a/L\right)-4{\left(a/L\right)}^{3}}.$$

where

(7) $$\xi =4\left(\frac{M_{\mathrm{cr}}}{M_{a}}\right)-3.$$

The meaning of a and L is the same as in Equation (2).

Equation (8) provides an estimate for the nominal flexural capacity (M_n_) of a BFRP-reinforced beam [29]:

(8) $$M_{n}=\{\begin{array}{c} \begin{array}{ccc} A_{f}f_{\mathrm{fu}}\left(d-\frac{\beta_{1}c}{2}\right) & \mathrm{if} & \frac{\rho_{f}}{\rho_{f,b}}\leq 1 \end{array}\\ \begin{array}{ccc} \rho_{f}f_{f}\left(1-0.59\frac{\rho_{f}f_{f}}{f_{c}^{\prime }}\right) & \mathrm{if} & \frac{\rho_{f}}{\rho_{f,b}}>1 \end{array} \end{array}.$$

In Equation (8), $A_{f}$ is the cross-sectional area of the FRP reinforcement, $f_{\mathrm{fu}}$ is the design tensile strength of the FRP material, d is the effective depth of the beam, $\beta_{1}$ is a stress-block factor for concrete, c is the height of the concrete compression face, $f_{c}^{\prime }$ is the specified compressive stress of concrete, and $f_{f}$ is the stress at the FRP rods

(9) $$f_{f}=\left(\sqrt{\frac{{\left(E_{f}\varepsilon_{\mathrm{cu}}\right)}^{2}}{4}+\frac{0.85\beta_{1}f_{c}^{\prime }}{\rho_{f}}E_{f}\varepsilon_{\mathrm{cu}}}-0.5E_{f}\varepsilon_{\mathrm{cu}}\right)\leq f_{\mathrm{fu}},$$

where $E_{f}$ is the elasticity modulus of the FRP rod; $\varepsilon_{\mathrm{cu}}=0.003$ stands for the ultimate compressive strain of concrete [28]; $\rho_{f}=A_{f}/\mathrm{bd}$, where b is the width of the cross-section of the beam; $\rho_{f,b}$ is the FRP reinforcement ratio at balanced strain condition.

## 4. Finite Element Model

### 4.1. Finite Elements and Mesh Generation

**Figure 4:**
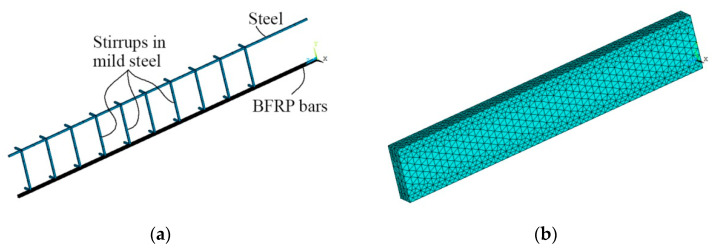
Finite element model of the BFRP-reinforced beam, generated finite element mesh: (**a**) longitudinal reinforcement in the compression zone (blue), stirrups (blue), and BFRP reinforcement (black); (**b**) concrete.

A detailed finite element model of the BFRP-reinforced beam is built using ANSYS mechanical APDL 19 (Figure 4). Pre-tensioned bars in BFRP, stirrups, and upper longitudinal reinforcement in mild steel are meshed with LINK180 finite element; concrete volumes are meshed with SOLID186 finite element. LINK180 is a uniaxial tension-compression element with two nodes and with three degrees of freedom at each node, i.e., translations in the nodal x, y, and z directions. SOLID186 is a 3D 20-node finite element, defined by 20 nodes, each node having three degrees of freedom per node, specifically, translations in the nodal x, y, and z directions. The finite element model contains a total of 2568 finite elements, i.e., 264 LINK180 finite elements and 2304 SOLID186 finite elements. Taking into consideration the symmetries of the beam and the quasi-static loading, a quarter of the structure is modeled using appropriate boundary conditions on the symmetry planes. The rolling support is modeled by restraining the vertical displacements of nodes at the support location (i.e., u_y_ = 0). The pre-tensioning of the BFRP tensile reinforcement is also modeled.

The load in the four-point bending test is incrementally applied. Stress and strain distributions in concrete are obtained by nonlinear static analysis for each increment of the applied load. The components of the stress and the strain tensors provide the input needed for the constitutive relation. Then, the damage variable is evaluated for each finite element, and the material properties of finite elements affected by damage are modified according to the pre-established constitutive relation. Finite elements of a critical level of damage (a predefined material constant) are deactivated.

### 4.2. Material Models

Constitutive relations are defined for all constituent materials (concrete, BFRP, and steel) and a failure criterion for concrete.

#### 4.2.1. Concrete

Constitutive relations are defined for all constituent materials (concrete, BFRP, and steel), and a failure criterion for concrete. The response of concrete is simulated by assuming a coupling between elasticity and damage, according to Equation (10) [30]:

(10) $$\sigma_{\mathrm{ij}}=\frac{\nu }{\left(1+\nu \right)\left(1-2\nu \right)}E\left(1-D\right)\varepsilon_{\mathrm{kk}}\delta_{\mathrm{ij}}+\frac{1}{\left(1+\nu \right)}E\left(1-D\right)\varepsilon_{\mathrm{ij}}.$$

where $\sigma_{\mathrm{ij}}$ and $\varepsilon_{\mathrm{ij}}$ are the components of the stress and the strain tensor, respectively; ν represents the Poisson’s ratio; E is the elasticity modulus of the undamaged concrete; $\varepsilon_{\mathrm{kk}}=\overset{3}{\underset{k=1}{\sum }}\varepsilon_{\mathrm{kk}}$ is the trace of the strain tensor; $\delta_{\mathrm{ij}}$ is the Kronecker delta; and D is the damage variable. 

The damage variable is assumed to depend on a set of model constants that should be identified through a comparison with experimental data. Within the model employed, the evolution of the damage variable depends on the equivalent strain [31] as:

(11) $$\varepsilon_{\mathrm{eqv}}=\sqrt{\sum_{j=1}^{3}\langle \varepsilon_{j}\rangle {}^{2}}.$$

where $\varepsilon_{j}$ are the eigenvalues of the strain tensor, and $\langle \varepsilon_{j}\rangle =0.5\left(\varepsilon_{j}+\left|\varepsilon_{j}\right|\right)$. 

When the damage variable reaches a specified, predefined value, a macroscopic crack is initiated in the corresponding location. The critical value of the damage variable is another model constant that should be identified against experimental data. In the identification process, the initial data contain the elasticity modulus of the undamaged material and the geometry of the test specimens. Then, strength requirements for the given concrete grade (in terms of compressive and tensile strength) are met based on tuning of the model constants. 

#### 4.2.2. BFRP

For the material model of the BFRP bar, an approximation is used by assuming material isotropy. This assumption is motivated by the choice of the finite element employed to mesh the BFRP reinforcement. The finite element LINK180 supports only axial tension/compression. The relevant material properties of BFRP are the elasticity modulus E_BFRP_ = 54 GPa and the Poisson’s ratio ν_BFRP_ = 0.23 [16,32,33,34].

#### 4.2.3. Steel

A predefined model for steel is used defining an initial elastic behavior followed by a hardening phase when yielding occurs [35,36]. However, it should be noted that presumably, in steel elements (i.e., longitudinal reinforcement in the compression face and stirrups), stresses do not reach the yield stress.

## 5. Numerical Results

**Figure 5:**
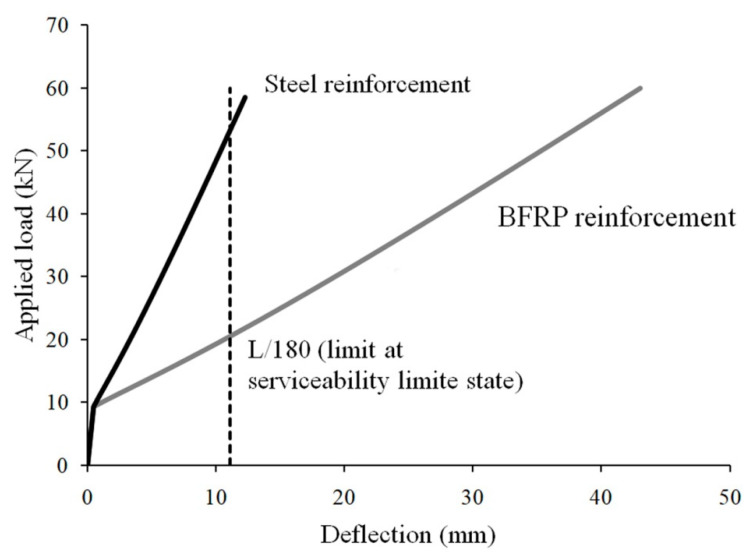
Simulation, based on [24] of the load-deflection response of a beam with tensile flexural reinforcement in mild steel (black line) and BFRP (grey line).

Figure 5 shows the results obtained by using the analytical model defined in Equations (1)–(9). As expected, because of the considerably lower elasticity modulus of the BFRP as compared with steel, the deflection of the beam reinforced with flexural BFRP reinforcement is significantly greater than the deflection of the beam with flexural steel reinforcement. In addition, for the conventional limit deflection $u_{\lim}=L/180$, where L is the span of the simply supported beam, the load-carrying capacity of the steel-reinforced beam is greater than that of the BFRP-reinforced beam. Tensile BFRP reinforcement is pre-tensioned to increase the performances of the BFRP-reinforced beam in terms of rigidity and load-carrying capacity without neglecting the serviceability limit states’ requirements [21].

The analytical model is comprehensive and a well-motivated model from a theoretical point of view. Validation through a comparison with empirical data has shown that the model accurately reproduces the experimental evidence, despite the slight overestimation of deflections for the lower moment level [37]. 

**Figure 6:**
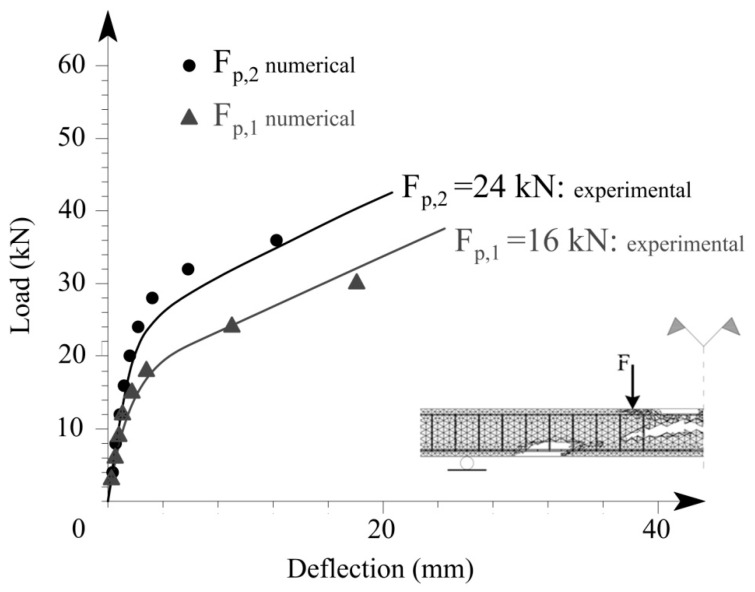
Load-deflection responses for various levels of pre-tensioning: pre-tensioning force equal to 11 kN (grey) and pre-tensioning force equal to 17 kN (black). Results obtained by finite element analysis are compared with experimental results.

It should be pointed out that analytical models do not capture the kinetics of the process of crack propagation. Crack propagation is considered to be an instantaneous process. The model contains only two states of the tension face of the beam, uncracked $\left(M_{a}<M_{\mathrm{cr}}\right)$ and cracked $\left(M_{a}\geq M_{\mathrm{cr}}\right)$. The analysis procedure based on the finite element method allows for the simulation of the incremental crack propagation via the successive deactivation of the concrete finite element where the local failure criterion is met. Figure 6 illustrates the results obtained by using an alternative approach, simulation of the BFRP-reinforced concrete beam based on a detailed finite element model. The numerical analysis predicts the response of BFRP-reinforced beans in terms of load-deflection relationships. It also predicts the potential failure mode (in Figure 6, concrete finite elements in which the critical value of the damage variable is reached are not displayed).

The positive effect of pre-tensioning on the overall behavior of the FRP-reinforced beam is apparent in Figure 6. Load-deflection relationships numerically obtained for two levels of pre-tensioning are shown: for pre-tensioning forces F_p,1_ = 16 kN or 20% of the BFRP rods load-carrying capacity, and F_p,2_ = 24 kN or 30% of the BFRP rods load-carrying capacity [21]. Figure 6 also shows experimental data provided by the same literature source.

Thus, pre-tensioning can compensate for the lower flexural rigidity of beams reinforced by flexural BFRP reinforcement as compared with traditional reinforced concrete beams. This conclusion agrees with previous results obtained by finite element simulation [38] and experimental testing of BFRP-reinforced concrete beams [21,22,39,40]. 

## 6. “Green” Basalt Fiber

**Figure 7:**
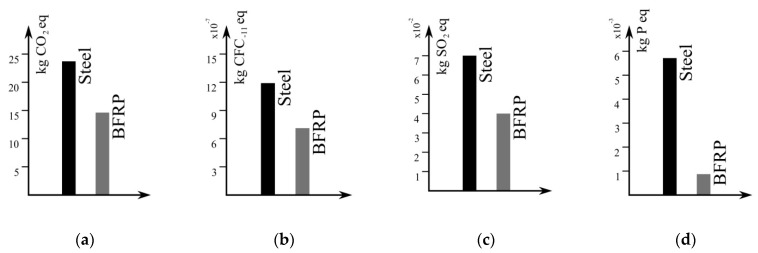
A comparison between the environmental markers (climate change emissions (**a**), ozone depletion (**b**), terrestrial acidification (**c**), and freshwater eutrophication (**d**)) for structural steel and BFRP [45].

Figure 7 depicts a comparison of the environmental impact of structural steel and BFRP based on reference values for four environmental marks, specifically, climate change emissions (Figure 7a), ozone depletion (Figure 7b), terrestrial acidification (Figure 7c), and freshwater eutrophication (Figure 7d). The embedded carbon (CO_2,eq_) is an indicator defined to assess possible climate-change emissions during the material production process. This indicator evaluates the carbon dioxide emitted during the whole production process and the in-use period. The monitored period might include the extraction of raw materials, their transport, refining, processing, assembly, and finally, their use, for example, as constituents of a structural element. The marker employed to assess effects resulting in ozone depletion is referred to as the relative level of CFC-11 (CFC_-11,eq_) emitted during the material production. Currently, it is widely accepted that ozone depletion due to man activities is mainly provoked by chlorofluorocarbons. Reportedly, the total amount of effective halogens (chlorine and bromine) in the atmosphere can be calculated [41]. In this context, chemical compounds are ranked in the function of the relative amount of degradation to the ozone layer they may cause. Terrestrial acidification is related to the depositions of nutrients such as nitrogen and sulfur. An increase in the acidifying nutrient concentration of soil is related to a decline in soil fertility. It may provoke an increase in plant tissue yellowing and seed germination failure. It may also trigger a decrease in new root production, leading to a reduction in photosynthetic rates and, thereby, a reduction in plant biomass. In extreme cases, this mechanism can provoke a decrease in the plants’ diversity [42,43,44]. To assess the sensibility of this mechanism to the production of a specific material, generally, it is suggested to monitor the quantity of sulfur dioxide (SO_2_). The marker used to quantify the influence of the production of a given material on freshwater eutrophication is the equivalent quantity of phosphorus (kg Peq). Eutrophication, most commonly driven by nitrogen and phosphorus, includes the growth of plants and algae and may result in oxygen depletion of the water. The comparison between steel and BFRP in terms of the above-mentioned environmental markers highlights the better performance of the latter material from an ecological perspective.

## 7. Conclusions

The comparison between the load-deflection responses of two geometrically identical beams, i.e., one with flexural steel reinforcement and the other with flexural BFRP reinforcement, outlines the lower flexural rigidity of the BFRP-reinforced beam. The numerical framework for this comparison is widely accepted, and reportedly, experimentally verified.

The finite element analysis has illustrated the positive effect of pre-tensioning on BFRP reinforcement, not taken into consideration the aforementioned model. The finite element analysis results show that the load-carrying capacity increases with an increase in the pre-tensioning force, confirming the experimental data found in the literature. Moreover, the finite element method-based algorithm allows for the modeling of a gradual propagation of damage and cracking in concrete. In addition to material models for BFRP and steel, the numerical FEM algorithm employs an original procedure defining a constitutive relation for concrete based on the continuum damage mechanics. A damage variable is plugged into the stress-strain relationship for concrete to account for damage accumulation. The deactivation of the finite elements in which the damage variable reaches a pre-established critical value simulates macroscopic cracking. Therefore, the model can predict the crack pattern at failure. 

As shown by the comparison between the environmental markers, the production and the implementation of BFRPs in construction, as a replacement for traditional steel reinforcement, reduces the negative impact on the environment. BFRP appears as an eco-friendly material that is in line with the concept of structural sustainability.

To summarize, BFRP appears as an attractive, eco-friendly material that, in some cases, will possibly enhance the sustainability of the civil engineering infrastructure, specifically in aggressive environments. On the one hand, reportedly, BFRPs possess many advantages: they are non-corrosive and non-toxic, of good magnetic insulation properties, are resistant to high temperature and high moisture conditions, are chemically stable, and support weathering tests well. On the other hand, some researchers report insufficient resistance to alkali in concrete. Regarding structural applications (compared to structural steel), BFRPs possess high tensile strength, but relatively low elasticity modulus, thus, providing a lower flexural rigidity to structural elements subjected to bending. Other critical aspects in the design and construction of concrete elements with internal BFRP reinforcement are the lower dowel action, the need for improvement of the bond behavior by implementing various types of coating, the need for complicated anchorage devices (for the pre-tensioned applications), and some difficulties in the fabrication of bent bars. In some countries, the lack of standards also impedes the application of BFRPs as a structural material, which is a situation requiring more intensive research in this field. 

Solutions to some issues inherent to currently available BFRP technology for construction have been discussed with respect to the background of design and analysis procedures for BFRP-reinforced structural elements. Further research should include simulation of benchmark experimental studies to validate the numerical procedure for various loading conditions. A possible implementation of the proposed algorithm requires checking the reproducibility of numerical results. A detailed finite element analysis would also provide an additional estimate of design code provisions.

## Data Availability

The study employs data available in publicly accessible repositories as well as data available in publicly accessible repositories that do not issue DOIs.
